# Characterization of Highly Pathogenic Avian Influenza Virus A(H5N6), Japan, November 2016

**DOI:** 10.3201/eid2304.161957

**Published:** 2017-04

**Authors:** Masatoshi Okamatsu, Makoto Ozawa, Kosuke Soda, Hiroki Takakuwa, Atsushi Haga, Takahiro Hiono, Aya Matsuu, Yuko Uchida, Ritsuko Iwata, Keita Matsuno, Masakazu Kuwahara, Toshiyo Yabuta, Tatsufumi Usui, Hiroshi Ito, Manabu Onuma, Yoshihiro Sakoda, Takehiko Saito, Koichi Otsuki, Toshihiro Ito, Hiroshi Kida

**Affiliations:** Hokkaido University, Sapporo Japan (M. Okamatsu, T. Hiono, K. Matsuno, Y. Sakoda, H. Kida);; Kagoshima University, Kagoshima, Japan (M. Ozawa, A. Matsuu);; University, Yamaguchi, Japan (M. Ozawa, A. Matsuu); Tottori University, Tottori, Japan (K. Soda, T. Usui, H. Ito, K. Otsuki, T. Ito);; Kyoto Sangyo University, Kyoto, Japan (H. Takakuwa, T. Yabuta, K. Otsuki);; Matsuoka Research Institute for Science, Koganei, Tokyo, Japan (M. Kuwahara);; National Agriculture and Food Research Organization, Tsukuba (Y. Uchida, T. Saito);; National Institute for Environmental Studies, Tsukuba, Japan (A. Haga, R. Iwata, M. Onuma)

**Keywords:** Highly pathogenic avian influenza virus, H5N6 subtype, viruses, influenza, Japan, zoonoses

## Abstract

Highly pathogenic avian influenza viruses (HPAIVs) A(H5N6) were concurrently introduced into several distant regions of Japan in November 2016. These viruses were classified into the genetic clade 2.3.4.4c and were genetically closely related to H5N6 HPAIVs recently isolated in South Korea and China. In addition, these HPAIVs showed further antigenic drift.

Since their emergence in ≈2010–11 in China (*1*), highly pathogenic avian influenza viruses (HPAIVs) that have the hemagglutinin (HA) genes of the H5 subtype classified into the genetic clade 2.3.4.4 have threatened global bird species, including wild birds and poultry, as well as humans. Although the H5 HA genes of these viruses are closely related, the subtypes of their neuraminidase (NA) genes vary widely. These new H5 HPAIVs with NA genes of various subtypes, the so-called H5Nx viruses, have spread globally, most likely because of their host preference for waterfowl, similar to the previous H5N1 HPAIVs (*2**–**4*). During the winter season 2014–15, H5N8 HPAIVs were isolated from wild birds and chickens in western Japan (*5**–**7*). In November 2016, HPAIVs of the H5N6 subtype were isolated in 3 geographically distant regions of Japan. We report the genetic and antigenic characteristics of 6 H5N6 HPAIVs.

## The Study

**Figure 1:**
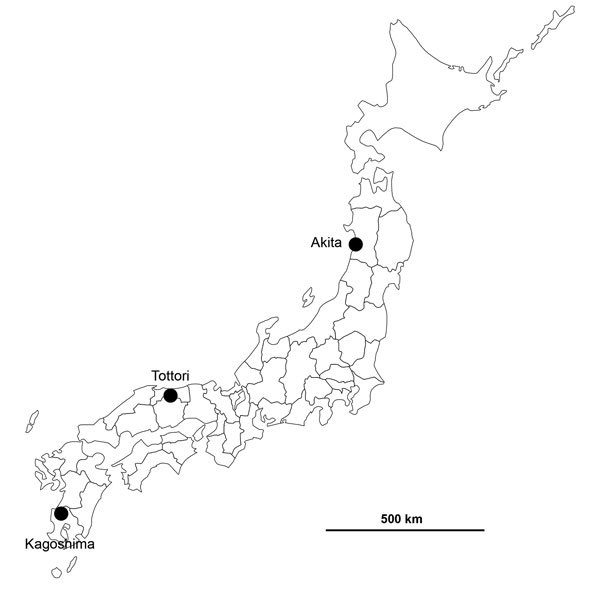
Locations of confirmed highly pathogenic avian influenza virus A(H5N6) infections in Akita, Tottori, and Kagoshima Prefectures, Japan, 2016.

The first suspected case of an HPAI outbreak in Japan during winter 2016–17 was reported from Akita Prefecture in northern Japan (Figure 1). A black swan (*Cygnus atratus*) in a zoo that died on November 15, 2016, tested positive for influenza virus antigen by a rapid diagnostic test. While this bird’s specimens underwent further analysis, another influenza virus was isolated from a water sample collected at an overwintering site of migratory birds in Kagoshima Prefecture at the southern tip of Japan on November 14, 2016 (Table 1). This isolate, A/environment/Kagoshima/KU-ngr-I/2016 (H5N6), was confirmed to be an H5N6 subtype having multiple basic amino acid residues, PLRERRRKR/GLF, at the cleavage site in the HA protein, which is characteristic of HPAIVs, by conventional reverse transcription PCR and Sanger sequencing. Subsequently, an isolate from the first black swan, A/black swan/Akita/1/2016 (H5N6), also was confirmed to be an H5N6 HPAIV, showing that all 3 chickens inoculated intranasally with 10^8.4^ of 50% egg infectious dose of the virus died within 2 days. In addition, a fecal sample of a common teal (*Anas crecca*) collected at an overwintering site of migratory birds in Tottori Prefecture in the middle of Japan on November 15, 2016, was reported to harbor an H5N6 HPAIV, A/teal/Tottori/1/2016 (H5N6) (Table 1). The isolation sites of these 3 H5N6 HPAIVs are distant (Figure 1), although the sample collection dates were close (Table 1). These 3 cases were followed by several reports of H5N6 HPAIVs, including A/black swan/Akita/2/2016 (H5N6), A/northern pintail/Tottori/b37/2016 (H5N6), and A/crane/Kagoshima/KU-4/2016 (H5N6), in Japan (Table 1). As of December 4, a total of 31 confirmed cases in wild birds had been reported to the Ministry of Environment (http://www.env.go.jp/nature/dobutsu/bird_flu/index.html), and 4 cases at poultry farms were confirmed in Japan (*8*).

**Table 1:** Details of highly pathogenic avian influenza virus A(H5N6) infections, Japan, November 2016

Date of report	Date of sample collection	Sample (species of bird)	Isolate
Nov 18	Nov 14	Water at an overwintering site	A/environment/Kagoshima/KU-ngr-I/2016 (H5N6)
Nov 21	Nov 15	Dead exhibition bird in a zoo (black swan)	A/black swan/Akita/1/2016 (H5N6)
Nov 21	Nov 6	Wild birds’ feces (northern pintail)	A/northern pintail/Tottori/b37/2016 (H5N6)
Nov 21	Nov 15	Wild birds’ feces (teal)	A/teal/Tottori/1/2016 (H5N6)
Nov 21	Nov 17	Dead exhibition bird in a zoo (black swan)	A/black swan/Akita/2/2016 (H5N6)
Nov 22	Nov 18	Dead wild bird (hooded crane)	A/crane/Kagoshima/KU-4/2016 (H5N6)

To clarify the genetic background of the H5N6 HPAIVs concurrently introduced into several distant regions of Japan, we determined the complete genome sequences of 5 of our isolates: A/black swan/Akita/1/2016 (H5N6) (GenBank/DDBJ/EMBL accession nos. LC198525–LC198532), A/teal/Tottori/1/2016 (H5N6) (GenBank/DDBJ/EMBL accession nos. LC199865–LC199872), A/northern pintail/Tottori/b37/2016 (H5N6) (GenBank/DDBJ/EMBL accession nos. LC200414-LC200421), A/environment/Kagoshima/KU-ngr-I/2016 (H5N6) (GISAID EpiFlu [http://platform.gisaid.org/], GenBank/DDBJ/EMBL accession nos. EPI861582–EPI861589), and A/crane/Kagoshima/KU-4/2016 (H5N6) (GenBank/DDBJ/EMBL accession nos. EPI867577–EPI867584) by Sanger and/or Illumina Miseq next-generation sequencing. These 5 isolates were almost genetically identical. Even among the HA genes, which are the most frequently mutated ones among the 8 gene segments, only 3–8 nt mutations, including 3 nonsynonymous mutations, were detected compared with the earliest strain, A/northern pintail/Tottori/b37/2016 (H5N6) (Technical Appendix Table). Thus, the 5 isolates would share a close common ancestor.

**Figure 2:**
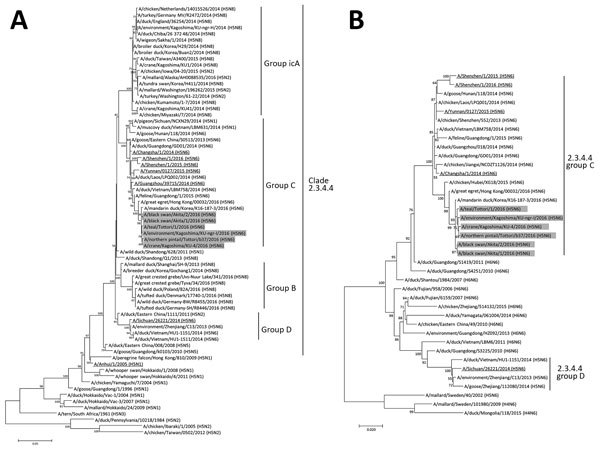
Phylogenetic trees of the HA and NA gene segments of highly pathogenic avian influenza virus A(H5N6) isolated in Japan. The nucleotide sequences of the H5 HA (A) and N6 NA (B) genes were analyzed by the maximum-likelihood method along with the corresponding genes of reference strains using MEGA 7.0 software (http://www.megasoftware.net/). Horizontal distances are proportional to the minimum number of nucleotide differences required to join nodes and sequences. Numbers at the nodes indicate the probability of confidence levels in a bootstrap analysis with 1,000 replications. Gray indicates viruses isolated in this study; underlining indicates viruses isolated in humans. The H5 HA gene sequences are classified into genetic clades as defined by Lee et al. (*9*). Scale bars indicate nucleotide substitutions per site. HA, hemagglutinin; NA, neuraminidase.

The phylogenetic tree analysis of the HA gene revealed that our isolates are classified into the genetic clade 2.3.4.4c and clustered with the recent H5N6 HPAIV isolates from wild and domestic birds and humans in China, in addition to an isolate South Korea, A/Mandarin duck/Korea/K16-187-3/2016 (H5N6) (Figure 2, panel A), on the basis of a recent classification in clade 2.3.4.4 (*9**,**10*). The NA genes of our isolates also form a single cluster together with the H5N6 HPAIV isolates from China into group C in the phylogenetic tree (Figure 2, panel B). In addition, the remaining 6 genes were genetically close to the recent H5N6 HPAIV isolates from China in the corresponding phylogenetic trees (Technical Appendix Figure 1), except for the polymerase basic 1 genes, which are most closely related to the counterpart of A/duck/Guangdong/S4040/2011 (H4N2) that was isolated from a domestic duck at a live bird market in China (*11*). Thus, the H5N6 HPAIV isolates would be derived from a reassortant that arose between an H5N6 HPAIV recently circulating in wild birds, poultry, or both in East Asia and in low pathogenicity avian influenza virus circulating in poultry in China. The genetic background of the H5N6 HPAIV isolates in this study is similar to the recent South Korea H5N6 virus collected in October 2016 and clearly different from that of recent H5Nx HPAIVs in Russia (*10*), Western European countries, and Alaska (*8*).

Our putative amino acid sequence comparison revealed that a leucine residue at position 134 in the HA protein (H3 numbering) was deleted, unlike that with the closest relative A/feline/Guangdong/1/2015 (H5N6) (Technical Appendix Figure 2). Our isolates have the amino acid sequence QQG at positions 226–228, which are located at the receptor-binding site in the HA protein, although the corresponding amino acid sequences of the previous H5 viruses are QSG or QRG (Technical Appendix Figure 2). These findings suggest that the receptor specificity of our H5N6 HPAIV isolates might be altered from their parental viruses (*12**,**13*). We also found 11 aa deletions in the stalk region of the NA protein, unlike that of A/duck/Vietnam/HU1–1151/2014 (H5N6), a representative virus strain of an N6 NA gene-based group D (Technical Appendix Figure 3), which belongs to a different cluster of the clade 2.3.4.4.

For HA antigenic characterization, we investigated the reactivity of chicken antiserum raised against several H5 isolates to our H5N6 HPAIV isolates using the hemagglutination inhibition test (*14*). We selected 1 reference virus strain, A/black swan/Akita/1/2016 (H5N6), and prepared single immunized chicken antiserum against the virus because of the limited variation of the nucleotide sequences in the HA genes among our 6 H5N6 HPAIV isolates. Antibody titer of antiserum of A/black swan/Akita/1/2016 (H5N6) were 16–32-fold higher against homologous virus than against the other strains (Table 2). The reactivity of the antiserum of A/chicken/Kumamoto/1-7/2014 (H5N8), whose HA gene belongs to the genetic clade 2.3.4.4, to A/black swan/Akita/1/2016 (H5N6) was 4-fold lower than that of the antiserum to the homologous combination. Moreover, none of the antiserum samples tested reacted strongly with A/black swan/Akita/1/2016 (H5N6) except for the homologous antiserum. These results indicate that the HA antigenicity of the H5N6 HPAIVs recently introduced in Japan differ appreciably from those of the previous H5Nx viruses.

**Table 2:** Antigenic analyses of H5 influenza viruses with antiserum*

Virus lineage/clade	Virus	Hemagglutination inhibition titers of the antiserum						
Mal/Hok (H5N1)	Ws/Hok (H5N1)	Pf/HK (H5N1)	Ck/Kum (H5N8)	B. swan/ Akita (H5N6)	Ck/Yam (H5N1)	Ck/Ibr (H5N2)
Eurasian								
–	Mal/Hok/24/2009 (H5N1)†	1,280	80	40	1,280	16	1,280	1,280
2.3.2.1	Ws/Hok/1/2008 (H5N1)	40	640	40	640	8	640	<20
2.3.4	Pf/HK/810/2009 (H5N1)	<20	20	2,560	20	8	80	<20
2.3.4.4 icA	Ck/Kumamoto/1-7/2014 (H5N8)	20	20	320	640	16	80	<20
2.3.4.4c	**B. swan/Akita/1/2016 (H5N6)**	<20	<20	80	160	256	80	<20
2.5	Ck/Yamaguchi/7/2004 (H5N1)	320	320	80	80	16	5,120	320
North American	Ck/Ibaraki/1/2005 (H5N2)	320	20	<20	<20	16	1,280	20,480

*Dash indicates virus does not belong to clade classification. Bold indicates virus isolated in this study. Underline indicates homologous titers. B. swan, black swan; Ck, chicken; HK, Hong Kong; Hok, Hokkaido; Mal, mallard; Pf, peregrine falcon; Ws, whooper swan. †Low pathogenic avian influenza viruses isolated from mallard (*15*) and chicken in Japan.

## Conclusions

We isolated 6 H5N6 HPAIVs from dead birds, fecal samples of migratory birds, and environmental water sample in 3 distant regions of Japan in November 2016. A genetic analysis showed that these isolates were genetically closely related to H5N6 HPAIVs recently isolated in China except for the polymerase basic 1 gene segment. The HA antigenicity of our H5N6 HPAIVs was demonstrated to have drifted further than viruses belonging to the same genetic clade 2.3.4.4. To prevent the spread of HPAIVs by wild birds, prompt elimination of HPAIVs is urgently needed in countries in Asia.

Technical Appendix Nucleotide and amino acid mutations in hemagglutinin (HA) genes of highly pathogenic avian influenza virus A(H5N6) isolates; phylogenetic trees of gene segments of isolates fromin Japan and reference strains; comparison of amino acid position 134 and 227 (H3 numbering) in H5 HA; comparison of amino acid sequence of neuraminidase stalk.

## References

[1] Smith GJ, Donis RO. World Health Organization/World Organisation for Animal Health/Food and Agriculture Organization (WHO/OIE/FAO) H5 Evolution Working Group. Nomenclature updates resulting from the evolution of avian influenza A(H5) virus clades 2.1.3.2a, 2.2.1, and 2.3.4 during 2013–2014. Influenza Other Respi Viruses. 2015;9:271–6. 10.1111/irv.12324 PMC4548997 25966311

[2] Claes F, Morzaria SP, Donis RO. Emergence and dissemination of clade 2.3.4.4 H5Nx influenza viruses-how is the Asian HPAI H5 lineage maintained. Curr Opin Virol. 2016;16:158–63. 10.1016/j.coviro.2016.02.005 26991931

[3] Global Consortium for H5N8 and Related Influenza Viruses. Role for migratory wild birds in the global spread of avian influenza H5N8. Science. 2016;354:213–7. 10.1126/science.aaf8852 27738169 PMC5972003

[4] Bi Y, Chen Q, Wang Q, Chen J, Jin T, Wong G, et al. Genesis, evolution and prevalence of H5N6 avian influenza viruses in China. Cell Host Microbe. 2016;20:810–21. 10.1016/j.chom.2016.10.022 27916476

[5] Ozawa M, Matsuu A, Tokorozaki K, Horie M, Masatani T, Nakagawa H, et al. Genetic diversity of highly pathogenic H5N8 avian influenza viruses at a single overwintering site of migratory birds in Japan, 2014/15. Euro Surveill. 2015;20:21132. 10.2807/1560-7917.ES2015.20.20.21132 26027484

[6] Usui T, Soda K, Tomioka Y, Ito H, Yabuta T, Takakuwa H, et al. Characterization of clade 2.3.4.4 H5N8 highly pathogenic avian influenza viruses from wild birds possessing atypical hemagglutinin polybasic cleavage sites. Virus Genes. 2016. 27738904 10.1007/s11262-016-1399-6

[7] Tanikawa T, Kanehira K, Tsunekuni R, Uchida Y, Takemae N, Saito T. Pathogenicity of H5N8 highly pathogenic avian influenza viruses isolated from a wild bird fecal specimen and a chicken in Japan in 2014. Microbiol Immunol. 2016;60:243–52. 10.1111/1348-0421.12369 26916882

[8] World Organization for Animal Health. Update on highly pathogenic avian influenza in animals (type H5 and H7) [cited 2016 Dec 4]. http://www.oie.int/en/animal-health-in-the-world/update-on-avian-influenza/2016/

[9] Lee DH, Bahl J, Torchetti MK, Killian ML, Ip HS, DeLiberto TJ, et al. Highly pathogenic avian influenza viruses and generation of novel reassortants, United States, 2014–2015. Emerg Infect Dis. 2016;22:1283–5. 10.3201/eid2207.160048 27314845 PMC4918163

[10] Lee DH, Sharshov K, Swayne DE, Kurskaya O, Sobolev I, Kabilov M, et al. Novel reassortant clade 2.3.4.4 avian influenza A(H5N8) virus in wild aquatic birds, Russia, 2016. Emerg Infect Dis. 2017;23:359–60. 10.3201/eid2302.161252 27875109 PMC5324796

[11] Jiao P, Cao L, Yuan R, Wei L, Song Y, Shen D, et al. Complete genome sequence of an H10N8 avian influenza virus isolated from a live bird market in Southern China. J Virol. 2012;86:7716. 10.1128/JVI.00959-12 22733881 PMC3416296

[12] Guo H, de Vries E, McBride R, Dekkers J, Peng W, Bouwman KM, et al. Highly pathogenic influenza A(H5Nx) viruses with altered H5 receptor-binding specificity. Emerg Infect Dis. 2017;23:220–31. 10.3201/eid2302.161072 27869615 PMC5324792

[13] Hiono T, Okamatsu M, Igarashi M, McBride R, de Vries RP, Peng W, et al. Amino acid residues at positions 222 and 227 of the hemagglutinin together with the neuraminidase determine binding of H5 avian influenza viruses to sialyl Lewis X. Arch Virol. 2016;161:307–16. 10.1007/s00705-015-2660-3 26542967 PMC5030063

[14] Hiono T, Ohkawara A, Ogasawara K, Okamatsu M, Tamura T, Chu DH, et al. Genetic and antigenic characterization of H5 and H7 influenza viruses isolated from migratory water birds in Hokkaido, Japan and Mongolia from 2010 to 2014. Virus Genes. 2015;51:57–68. 10.1007/s11262-015-1214-9 26036326

[15] Yamamoto N, Sakoda Y, Motoshima M, Yoshino F, Soda K, Okamatsu M, et al. Characterization of a non-pathogenic H5N1 influenza virus isolated from a migratory duck flying from Siberia in Hokkaido, Japan, in October 2009. Virol J. 2011;8:65. 10.1186/1743-422X-8-65 21310090 PMC3048565

