# Supplementary material for: Characterization of Highly Pathogenic Avian Influenza Virus A(H5N6), Japan, November 2016
Source: Emerg Infect Dis. 2017 Apr;23(4):691–5. doi: 10.3201/eid2304.161957 (PMC5367431; doi:10.3201/eid2304.161957)
Supplement: Technical Appendix — Nucleotide and amino acid mutations in hemagglutinin (HA) genes of highly pathogenic avian influenza virus A(H5N6) isolates; phylogenetic trees of gene segments of isolates fromin Japan and reference strains; comparison of amino acid position 134 and 227 (H3 numbering) in H5 HA; comparison of amino acid sequence of neuraminidase stalk. [file 16-1957-Techapp-s1.pdf]

Article DOI: http://dx.doi.org/10.3201/eid2304.161957

Characterization of Highly Pathogenic Avian Influenza Virus A(H5N6), Japan, November 2016

Technical Appendix

Technical Appendix Table. Nucleotide and amino acid mutations in hemagglutinin genes of highly pathogenic avian influenza virus A(H5N6) isolates*

	Nucleotide position of hemagglutinin gene													
Virus	31	33	57	202	208	339	516	520	608	652	658	1125	1527	Mutation
A/northern pintail/Tottori/b37/2016 (H5N6)	G	T	T	T	G	C	T	A	C	A	G	G	G	–
A/environment/Kagoshima/KU-ngr-I/2016 (H5N6)	T	·	·	·	·	·	C	·	·	T	T	A	·	5
A/black swan/Akita/1/2016 (H5N6)	·	C	C	C	·	·	C	·	T	T	T	·	A	8
A/teal/Tottori/1/2016 (H5N6)	·	·	·	·	·	T	C	C	·	T	T	·	·	5
A/crane/Kagoshima/KU-4/2016 (H5N6)	·	·	·	·	A	·	·	·	·	T	T	·	·	3
	Amino acid position of hemagglutinin (H3 numbering)													
Virus	(11)†		–	–	63	–	–	163	192	207	209	–	–	Mutation
A/northern pintail/Tottori/b37/2016 (H5N6)	V		I	L	D	D	D	T	T	T	V	G	E	–
A/environment/Kagoshima/KU-ngr-I/2016 (H5N6)	F		·	·	·	·	·	·	·	S	L	·	·	3
A/black swan/Akita/1/2016 (H5N6)	·		·	·	·	·	·	·	I	S	L	·	·	3
A/teal/Tottori/1/2016 (H5N6)	·		·	·	·	·	·	P	·	S	L	·	·	3
A/crane/Kagoshima/KU-4/2016 (H5N6)	·		·	·	N	·	·	·	·	S	L	·	·	3
Character of the location	Signal peptide				Bottom of head			Surface of head	190-helix	Trimeric interface				

*Dot (・) indicates this nucleotide/amino acid is same as that of A/northern pintail/Tottori/b37/2016 (H5N6). Dash indicates no amino acid change in the position.

†Numbering from the initiating methionine in H5 hemagglutinin.

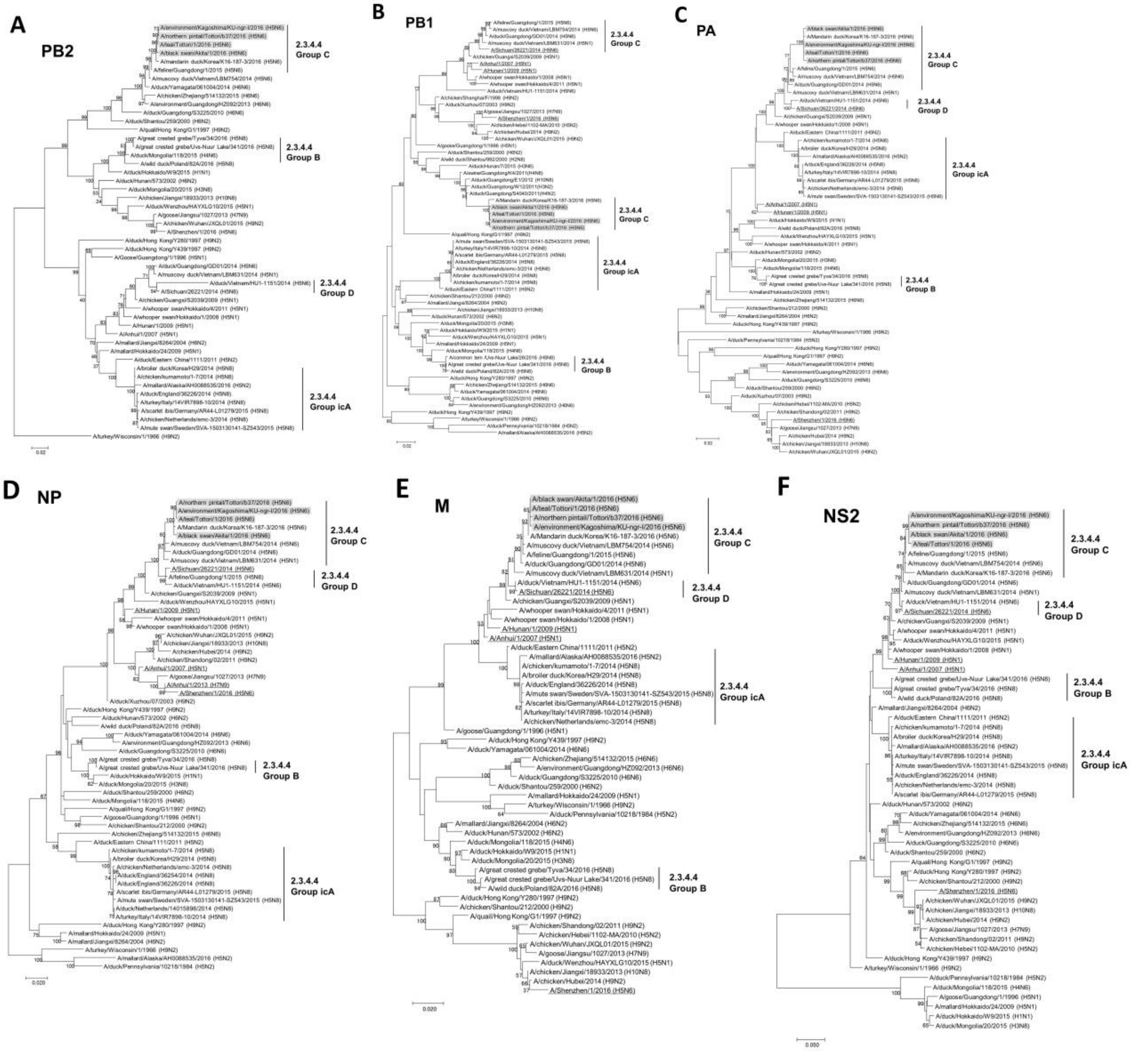

Technical Appendix Figure 1. Phylogenetic trees of the PB2 (A), PB1 (B), PA (C), NP (D), M (E), and NS (F) gene segments of highly pathogenic avian influenza virus A(H5N6) isolated in Japan during November 2016 and reference strains. Sequences were analyzed by the maximum-likelihood method along with the corresponding genes of reference strains by using MEGA 7.0 software (http://www.megasoftware.net/). Horizontal distances are proportional to the minimum number of nucleotide differences required to join nodes and sequences. Digits at the nodes indicate the probability of confidence levels in a bootstrap analysis with 1,000 replications. The viruses isolated in this study are highlighted in gray. The viruses

isolated in humans are underlined. Scale bars indicate nucleotide substitutions per site. M, matrix; NP, nucleoprotein; NS, nonstructura; PA, polymerase acidic; PB1, polymerase basic 1; PB2, polymerase basic 2.

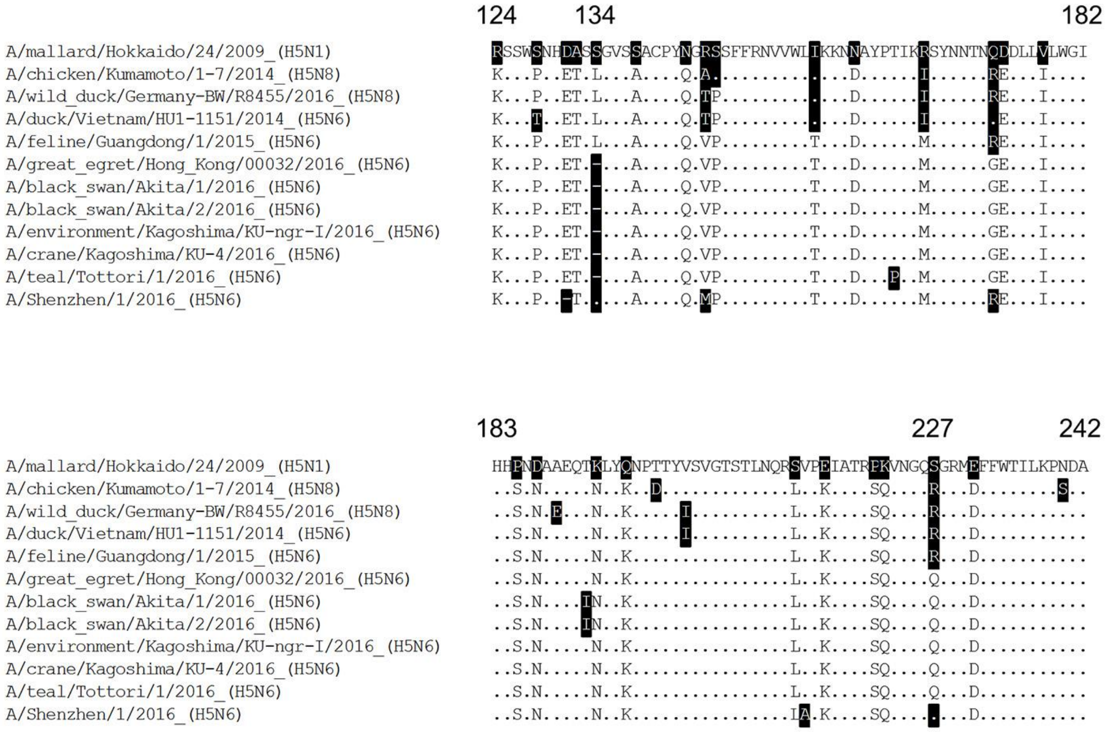

Technical Appendix Figure 2. Comparison of amino acid position 134 and 227 (H3 numbering) in H5 hemagglutinin (HA). A leucine residue at position 134 was deleted in highly pathogenic avian influenza virus A(H5N6) isolated in Japan during November 2016, compared with the closest relative A/feline/Guangdong/1/2015 (H5N6). The amino acid sequence QQG at positions 226–228, which are located at the receptor-binding site in the HA protein, although the corresponding amino acid sequences of the previous H5 viruses are QSG or QRG.

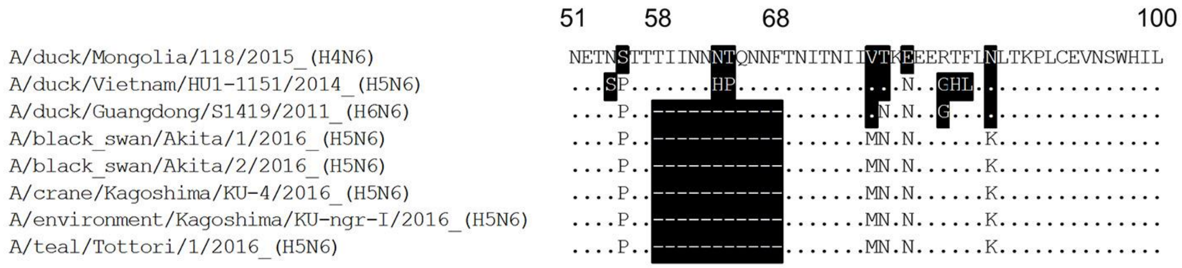

Technical Appendix Figure 3. Comparison of amino acid sequence of neuraminidase (NA) stalk. Eleven amino acid deletions (58–68) in the stalk region of the NA protein compared with A/duck/Vietnam/HU1–1151/2014 (H5N6), a representative virus strain of an N6 NA gene-based group D.
